# Regulation of the NLRP3 Inflammasome by Post-Translational Modifications and Small Molecules

**DOI:** 10.3389/fimmu.2020.618231

**Published:** 2021-02-02

**Authors:** Jin Kyung Seok, Han Chang Kang, Yong-Yeon Cho, Hye Suk Lee, Joo Young Lee

**Affiliations:** BK21 PLUS Team, College of Pharmacy, The Catholic University of Korea, Bucheon, South Korea

**Keywords:** innate immunity, pattern-recognition receptors, cell signaling, inflammation, pharmacological inhibitor

## Abstract

Inflammation is a host protection mechanism that eliminates invasive pathogens from the body. However, chronic inflammation, which occurs repeatedly and continuously over a long period, can directly damage tissues and cause various inflammatory and autoimmune diseases. Pattern recognition receptors (PRRs) respond to exogenous infectious agents called pathogen-associated molecular patterns and endogenous danger signals called danger-associated molecular patterns. Among PRRs, recent advancements in studies of the NOD-, LRR- and pyrin domain-containing protein 3 (NLRP3) inflammasome have established its significant contribution to the pathology of various inflammatory diseases, including metabolic disorders, immune diseases, cardiovascular diseases, and cancer. The regulation of NLRP3 activation is now considered to be important for the development of potential therapeutic strategies. To this end, there is a need to elucidate the regulatory mechanism of NLRP3 inflammasome activation by multiple signaling pathways, post-translational modifications, and cellular organelles. In this review, we discuss the intracellular signaling events, post-translational modifications, small molecules, and phytochemicals participating in the regulation of NLRP3 inflammasome activation. Understanding how intracellular events and small molecule inhibitors regulate NLRP3 inflammasome activation will provide crucial information for elucidating the associated host defense mechanism and the development of efficient therapeutic strategies for chronic diseases.

## Activation of the NLRP3 Inflammasome

NLRP3 is an intracellular sensor in the NLRP3 inflammasome that recognizes the widest range of pathogen-associated molecular patterns (PAMPs) and danger-associated molecular patterns (DAMPs) among NLRP family members. NLRP3 has three domains: an amino-terminal pyrin domain (PYD) that binds to ASC, a NACHT domain with ATPase activity, and an LRR domain that induces autorepression by folding back onto the NACHT domain (1). The ATPase activity of the NACHT domain in particular has been studied as a therapeutic target for NLRP3-related diseases (1).

ASC is an adapter protein that acts as a bridge between NLRP3 and caspase-1. ASC is a bipartite complex consisting of a PYD domain that interacts with NLRP3 and a CARD domain that interacts with caspase-1. The PYD domain of ASC is also required for its self-association as well as its interaction with NLRP3 (2).

Caspase-1 is a cysteine ​​protease that is synthesized as a zymogen and is capable of processing members of the interleukin-1 (IL-1) family, such as IL-1β and IL-18 (3). Full-length caspase-1 has three domains: an amino-terminal CARD, a central large catalytic domain (p20) and a carboxy-terminal small catalytic subunit domain (p10) (4). Caspase-1 clusters on the ASC and is self-cleaving at the linker between p20 and p10, resulting in a complex of p33 (including CARD and p20) and p10. Caspase-1 remains bound to ASC and exhibits proteolytic activity, while additional processing between CARD and p20 releases p20 and p10 from the ASC (5). The released p20–p10 heterotetramer is unstable in the cell and terminates its protease activity (6).

Canonical NLRP3 inflammasome activation requires a priming step. The priming step triggers upregulation of NLRP3 and IL-1β gene as well as post-translational licensing of NLRP3 inflammasome. Priming is initiated by Toll-like receptor (TLR) activation and cytokines such as TNF and IL-1β. Through this process, NF-κB, a transcription factor, is activated and transcriptionally upregulates NLRP3 and pro IL-1β. Moreover, priming sets up NLRP3 to form inflammasome assembly (7) or rescues from degradation (8) by licensing the proteins to form the correct morphology for self-oligomerization and interaction with ASC, through various post-translational modifications (PTM) to NLRP3. PTMs include ubiquitylation, deubiquitination, phosphorylation, and sumoylation of NLRP3 (7). The PTMs of NLRP3 occur during processes such as priming, activation, and resolution. Thus, priming signals regulate NLRP3 inflammasome activation through transcription-dependent pathways and PTMs.

After priming, NLRP3 responds to activating stimuli and assembles the NLRP3 inflammasome complex. These stimuli are derived from PAMPs during pathogen infections or DAMPs released from damaged host cells and include bacterial or viral pathogens, fungi, ATP, pore-forming toxins, crystalline substances, nucleic acids, and hyaluronan. The mechanisms of NLRP3 inflammasome activation that occur in response to various stimuli include the efflux of potassium, the secretion of cathepsin into the cytoplasm following lysosome degradation, the translocation of NLRP3 to the mitochondria, the production of free radicals in the mitochondria, and the secretion of mitochondrial DNA or cardiolipin. Once stimulated, NLRP3 is oligomerized by homotypic interactions in the NACHT domains (9). Subsequently, oligomerized NLRP3 recruits ASC through PYD-PYD interactions and creates multiple helical ASC filaments that combine with ASC speck, a single macromolecule (10). Assembled ASC recruits caspase-1 through CARD-CARD interactions and activates caspase-1 through its self-cleavage (6). Then, the activated NLRP3 inflammasome hydrolyzes inactive pro-caspase-1 to activate caspase-1, and active caspase-1 then induces the production and secretion of inflammasome-specific cytokines such as IL-1β and IL-18 while simultaneously inducing pyroptosis, which is inflammatory cell death (11).

Pyroptosis is an inflammatory form of lytic programmed cell death that is activated by NLRP3 inflammasomes. Interestingly, a recent study showed that GSDMD is a crucial mediator of pyroptosis (12). The amino-terminal cell death domain of GSDMD (GSDMD^N-term^) possesses a central short linker region and a carboxy-terminal autoinhibition domain (13). Caspase-1 cleaves and releases GSDMD from the carboxyl terminus to overcome intramolecular inhibition, after which GSDMD^N-term^ combines with phosphatidylinositol phosphates and phosphatidylserine in the inner leaflet of the cell membrane and oligomerizes. This oligomer is then inserted into the plasma membrane to form 10-14 nm pores containing 16 symmetrical protomers for cell killing (14, 15). Additionally, GSDMD^N-term^ exhibits bactericidal activities by combining with cardiolipin, which is present in the internal and external bacterial membranes (14, 16). Cardiolipin is also present on the internal and external mitochondrial membranes following NLRP3 activation (17). However, it is unclear whether GSDMD^N-term^ penetrates the mitochondria to combine with mitochondrial cardiolipin.

**Figure 1:**
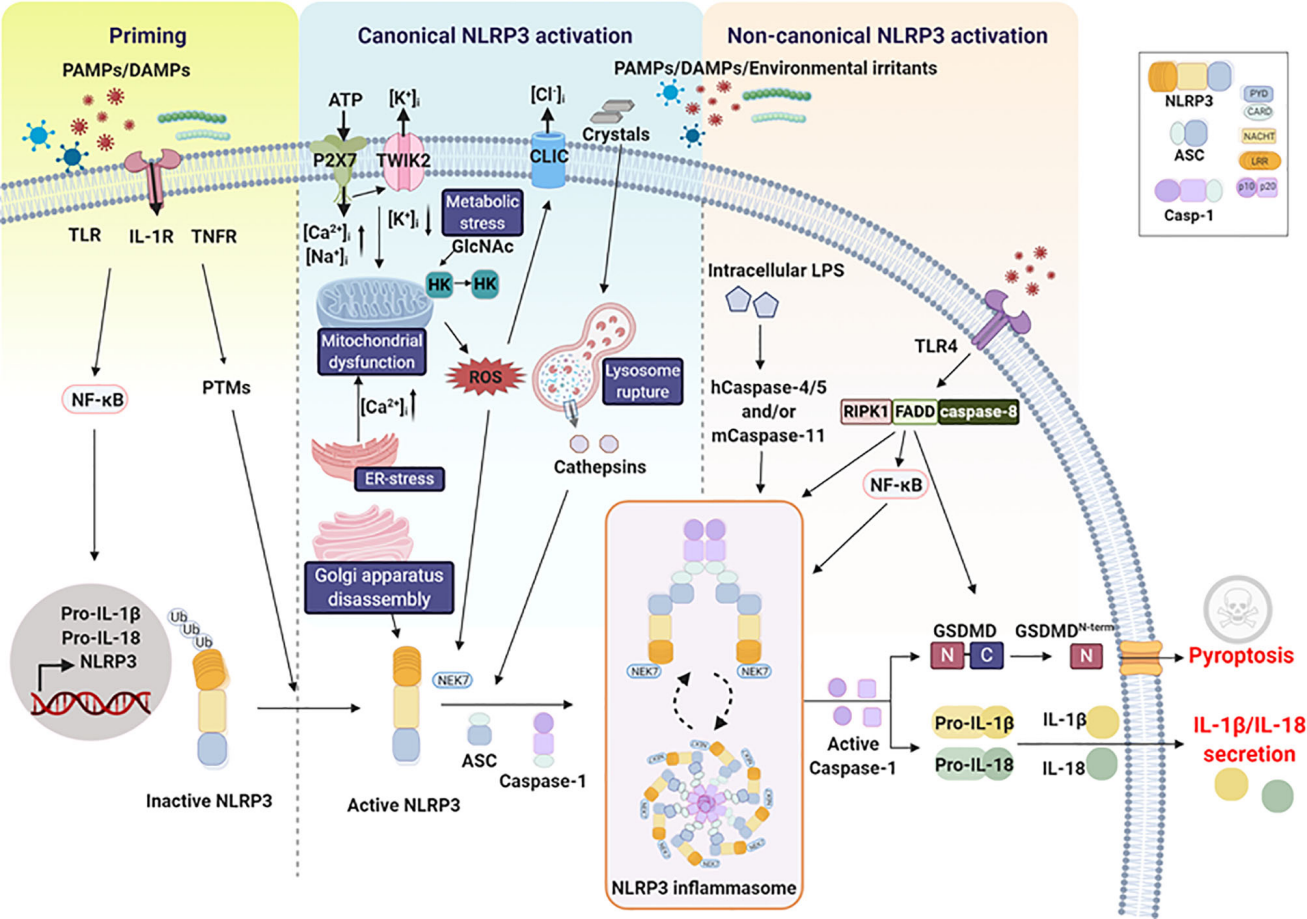
NLRP3 inflammasome pathway. Two steps are required for NLRP3 inflammasome activation. The first step is priming, which is triggered by microbial molecules or endogenous cytokines and is required to induce the expression of pro-interleukin-1β (IL-1β), pro-IL-18, and NLRP3 *via* the activation of the transcription factor nuclear factor-κB (NF-κB). The second step is NLRP3 inflammasome activation, which includes canonical and noncanonical activation pathways and is induced by a number of PAMPs and DAMPs. The canonical activation pathway involves stimulation-mediated activation signals such as ion fluxes, lysosome rupture, mitochondrial dysfunction, Golgi apparatus disassembly, metabolic stress, and ER stress. Activation of the inflammasome causes caspase-1 activation, leading to the maturation and release of IL-1β/IL-18 and pyroptosis. The noncanonical activation pathway is mediated by human caspase-4, human caspase-5, and mouse caspase-11, indirectly promoting the production of pro-IL-1β or pro-IL-18. Receptor-interacting protein kinase 1 (RIPK1), FAS-associated death domain protein (FADD), and caspase-8 are involved in this pathway by regulating NF-κB activation, leading to NLRP3 inflammasome activation.

Full activation of the NLRP3 inflammasome is accomplished by a well-concerted mechanism which includes the priming step and the activation step (**Figure 1**). The priming is an essential prerequisite to induce expression of NLRP3 and pro-form of IL-1β and IL-18 while the activation step involves an inflammasome assembly in an organized fashion to make pro-caspase-1 activated. The combination of these two events is expected to commonly take place when the host is exposed to PAMPs and DAMPs since PAMPs and DAMPs can activate both priming and activation steps.

## Noncanonical and Alternative Pathways for NLRP3 Inflammasome Activation

Recently, caspase-4 and -5 in human and caspase-11 in mouse have been shown to indirectly promote pro-IL-1β or pro-IL-18 activation by inducing NLRP3 inflammasome activation as a noncanonical NLRP3 activation pathway (18, 19). The noncanonical pathway is initiated by direct binding of these caspases to intracellular LPS (iLPS) produced by Gram-negative bacteria, independent of TLR4, the conventional LPS receptor (20). Caspase-11 induces extracellular release of ATP, which in turn activates P2X7 receptor and induces K^+^ efflux, leading the activation of NLRP3 inflammasome and production of mature IL-1β (21). In addition, activated caspase-4, -5, and -11 cleave gasdermin D (GSDMD), resulting in pyroptosis (13, 16). Caspase-4, -5, and -11 initiate pyroptosis similarly to caspase-1, but they do not directly cleave pro-IL-1β or pro-IL-18 (22).

Caspase-8, the apical activator caspase, provides an alternative pathway of the NLRP3 inflammasome activation, culminating in IL-1β and IL-18 maturation as well as cell death (23, 24). Notably, caspase-8-dependent NLRP3 activation is a species-specific pathway that is present in human and porcine peripheral blood mononuclear cells (PBMCs), but not murine cells (25). TLR4 stimulation by PAMPs and/or DAMPs activates RIPK1-FADD-caspase-8 signaling, which can directly trigger canonical NLRP3 oligomerization and inflammasome assembly as well as facilitate NF-κB transcription (25). In addition, RIPK1-FADD-caspase-8 signaling is involved in TLR3 priming for activation of NLRP3 inflammasome (26). Blockade of TGF-β activated kinase-1 (TAK1) by the *Yersinia* bacteria leads to cleavage of GSDMD RIPK1- and caspase-8-dependently, promoting the NLRP3 inflammasome activation and IL-1β secretion as well as cell death (27). In contrast, a negative role of caspase-8 in regulation of NLRP3 inflammasome was reported (28). Caspase-8-deficient dendritic cells showed higher production of IL-1β with enhanced activation of NLRP3 inflammasome, which is dependent on the functions of RIPK1 and RIPK3 (28). Although the exact role of caspase-8 in the regulation of NLRP3 inflammasome and inflammatory processes remains to be determined, the studies suggest the possible link between NLRP3 inflammasome, apoptosis, and inflammation mediated by caspase-8.

Considering complexity of *in vivo* inflammatory processes, noncanonical and alternative pathways for the NLRP3 inflammasome activation in addition to canonical pathway may play differential roles in the pathology of inflammatory diseases in different context.

## Intracellular Events Regulating NLRP3 Inflammasome Activation

### Ion Fluxes

Most NLRP3 stimuli, including ATP, nigericin, and particulate matter induce K^+^ efflux (29). The P2X7 receptor, which belongs to the P2X subfamily of ligand-gated ion channels with purine P2 receptors, can be activated by high concentrations of extracellular ATP, leading to the,NLRP3 inflammasome activation (30). The P2X7 receptor plays an important role in mediating the innate immune response by regulating the expression of pro-inflammatory cytokines of the IL-1 family (31). After ATP stimulation, P2X7 promotes Ca^2+^ and Na^+^ influx and coordinates with the K^+^ channel, two-pore domain weakly inward rectifying K^+^ channel 2 (TWIK2), which mediates K^+^ efflux (32). Lowering cytoplasmic K^+^ concentration was sufficient to activate the NLRP3 inflammasome (33). Active caspase-11 led to a decrease of intracellular potassium K^+^ levels, resulting in the activation of the NLRP3 inflammasome, suggesting the link between noncanonical and canonical NLRP3 activation pathways (33). K^+^ efflux is also suggested as the mechanism for which cytopathogenic RNA viruses such as vesicular stomatitis virus (VSV) or encephalomyocarditis virus (EMCV) induce activation of the NLRP3 inflammasome (34). These cytopathogenic viruses triggered a lytic cell death, which led to K^+^ efflux (34).

Na^+^ influx plays a regulatory role in NLRP3 inflammasome activation, possibly by regulating stimulus-induced K^+^ efflux. Combination of K^+^ efflux with Na^+^ influx is considered necessary for activation of the NLRP3 inflammasome induced by crystalline and lysosomal destabilization (35). Overactivation of the epithelial Na^+^ channel, ENaC, aggravated NLRP3 inflammasome activation by enhancing Na^+^ influx and subsequently K^+^ efflux, culminating in the exacerbated inflammatory responses in cystic fibrosis (36). High salt treatment increased production of IL-1β in monocytes and dendritic cells while treatment with an ENaC inhibitor, amiloride, blocked IL-1β production in these cells (37). Similarly, high salt diet to mice induced increased expression of NLRP3 and pro‐IL-1β in monocytes and dendritic cells whereas treatment with amiloride, an ENaC inhibitor, to mice fed high salt diet showed less expression of NLRP3 and pro‐IL-1β, suggesting an ENaC-dependent activation of NLRP3 inflammasome in response to high salt (37).

The importance of chloride efflux in regulation of NLRP3 inflammasome activation has been noted and the involvement of the volume regulated anion channel (VRAC) was reported (38, 39). Knockout of LRRC8A, a subunit of VRAC, in macrophages resulted in significant impairment of ASC oligomerization, caspase-1 cleavage, and IL-1β processing induced by hypotonicity, showing the critical role of VRAC in the regulation of NLRP3 inflammasome activation (40). Chloride intracellular channels (CLICs) have been reported to regulate NLRP3 inflammasome activation (41). A nonsteroidal anti-inflammatory agent inhibited chloride leakage through VRAC to prevent NLRP3 inflammasome activation (42) and CLIC was suggested to function as a VRAC activator (43). Various Cl^-^ channel inhibitors, including 4,4’-diisothiocyano-2,2’stilbene-disulfonic acid (DIDS) (43), 5-nitro-(3-phenylpropylamino) benzoic acid (NPPB) (38), flufenamic acid, mefenamic acid (42), and indanyloxyacetic acid 94 (IAA94) (43), block the NLRP3 inflammasome but cannot block the NLRC4 or AIM2 inflammasomes. CLICs translocate to the plasma membrane and promote NLRP3-NEK7 interactions, triggering Cl^-^ flux as a downstream event of mitochondrial dysfunction that regulates NLRP3 inflammasome activation (43). Cl^-^ efflux can induce ASC speck formation but does not induce NLRP3 inflammasome activation without K^+^ efflux (44). Further studies are needed to elucidate how Cl^-^ flux coordinates with other ionic events to trigger NLRP3 inflammasome activation.

### Lysosomal Destabilization

The phagocytosis of particulates causes lysosomal rupture, releasing particulates into the cytoplasm. Lysosomal destabilization was first revealed as a pathway mediating the activation of NLRP3 inflammasome by amyloid β, a pathogenic misfolded protein expressed in Alzheimer’s disease (45). The accumulation of crystals such as β-amyloid, monosodium urate (MSU), silica, and asbestos, in the cell destabilizes phagosomes and leads to the release of various components, including proteases, lipases, cathepsins, and Ca ^2+^ in the cytosol, leading to K^+^ efflux and the induction of NLRP3 assembly and activation (46). In addition, in Prion disease, the prion protein (PrP) misfolds induced lysosome destabilization and NLRP3 inflammasome activation (47). Lysosome rupture and the release of lysosomal hydrolases, especially cathepsin B, have been shown to be essential for albumin-induced tubulointerstitial inflammation (TI) and fibrosis, suggesting that lysosomal damage is involved in the pathogenesis of chronic kidney disease through NLRP3 inflammasome activation (48). Anti-cancer chemotherapeutic agents such as gemcitabine and 5-fluorouracil, induced activation of the NLRP3 inflammasome, which was dependent on lysosomal permeabilization and the release of cathepsin B, while this activation in myeloid-derived suppressor cells blunted their anticancer efficacy (49). In addition to cathepsin B, multiple cathepsins such as cathepsin L, C, S, and X had been shown to promote both pro-IL-1β synthesis and NLRP3 activation in compensatory and independent manners (50, 51). Lysosome destabilization is linked to ion flux in the process of NLRP3 inflammasome activation (29). The lysosome-destabilizing agonist, Leu-Leu-O-methyl ester (LLME), induced lysosome membrane permeabilization, which correlated with K+ efflux and NLRP3 inflammasome activation in murine dendritic cells (52). However, extensive lysosome destabilization attenuated NLRP3 inflammasome activation with increase of Ca^2+^ influx, while it potentiated necrosis in murine bone marrow-derived dendritic cells (53).

Lysosomal destabilization-induced activation of the NLRP3 inflammasome is the feature of particulate matter and crystalline, elucidating the pathological mechanisms of the relevant inflammatory diseases, including amyloid β with Alzheimer’s disease, monosodium urate with gout, cholesterol crystalline with atherosclerosis, silica with silicosis, and asbestos with asbestosis.

### Mitochondrial Dysfunction

Cardiolipin (1,3-bis(*sn*-3’-phosphatidyl)-*sn*-glycerol), a phospholipid constituent of the inner mitochondria membrane, links the mitochondria to the NLRP3 inflammasome activation (17). During mitochondrial stress, cardiolipin is exposed to the outer membrane, where it serves as a binding site during autophagy and apoptosis (54). Cardiolipin independently interacts with NLRP3 and full-length caspase-1, of which event is essential for inflammasome activation. MAVS is a mitochondrial protein that is necessary for the post-stimulus activation of the NLRP3 inflammasome by functioning as an adaptor protein in RNA-sensing pathways associated with RNA virus infections (55, 56). MAVS promotes NLRP3 inflammasome activation by recruiting NLRP3 to the mitochondrial outer membrane (55). Although MAVS is necessary for the RNA virus infection-mediated activation of the NLRP3 inflammasome, it may be dispensable for NLRP3 activation by other stimuli. Mitofusin 2 (Mfn 2), which is present in the outer mitochondrial membrane, endoplasmic reticulum, and contact sites of mitochondria-associated membrane, has been reported to be an essential factor for NLRP3 activation during RNA virus infection (57). During viral infection, Mfn2 forms a complex with MAVS and aids in the localization of NLRP3 in the mitochondria (57).

Reactive oxygen species (ROS) production was involved in the activation of NLRP3 inflammasome induced by ATP, MSU crystal, silica, and asbestos, suggesting mitochondrial ROS as a critical mediator for the NLRP3 inflammasome activation (58). Caspase-1 activation and IL-1β production in lipopolysaccharide (LPS)- and ATP-treated macrophages are dependent on mtROS generation and mitochondrial membrane permeability transition (59). The translocation of mitochondrial DNA (mtDNA) to cytosol was correlated with the activation of caspase-1 activation in LPS- and ATP-treated macrophages (59). Furthermore, oxidized mtDNA released during programmed cell death induced by NLRP3 activators such as ATP, bound to and activated the NLRP3 inflammasome (60). The *de novo* synthesis of mtDNA was induced by TLR signals accompanied with an expression of CMPK2, an enzyme that provides deoxyribonucleotides for mtDNA synthesis (61). CMPK2-dependent mtDNA synthesis resulted in the production of oxidized mtDNA fragments was required for NLRP3 inflammasome activation (61). The role of mtDNA-mediated activation of NLRP3 inflammasome in the development of inflammatory diseases is reported with Type 1 Diabetes (62).

These suggest mitochondria as central regulators of NLRP3 inflammasome activation induced by cellular stress, infections, and the NLRP3 activators, accompanying with mitochondrial dysfunction to promote the activation of NLRP3 inflammasome.

### Golgi Apparatus Disassembly

NLRP3 stimuli have been shown to promote the disassembly of the trans-Golgi network into vesicles called the dispersed trans-Golgi network (dTGN) using a cellular reconstitution system. The phospholipid phosphatidylinositol-4-phosphate of dTGN recruits NLRP3 and promotes its aggregation, which is essential for downstream ASC oligomerization and caspase-1 activation (63). The K^+^ efflux-dependent stimulus (i.e., nigericin) and K^+^ efflux-independent stimulus (i.e., imiquimod) aid in the formation of dTGN and cause NLRP3 aggregation. However, K^+^ efflux, is only necessary for the recruitment of NLRP3 and not for dTGN formation (64), indicating that the K^+^ efflux-dependent and mitochondria-dependent activation of NLRP3 are two separate pathways that converge at the Golgi disassembly stage.

### Metabolic Stress

Glucose phosphorylation, the first step in glycolysis, is mediated by hexokinase. During bacterial infection, the decomposition of the bacterial cell wall component peptidoglycan in lysosomes releases N-acetylglucosamine (GlcNAc). Hexokinase, which is located on the mitochondrial membrane, then combines with GlcNAc and promotes its relocalization in the cytosol. This GlcNAc-induced hexokinase relocalization promotes NLRP3 inflammasome activation regardless of K^+^ efflux (65). Although chemical disruption of glycolysis activates the NLRP3 inflammasome following priming (66), the interpretation of such observations is complicated, as the inhibition of glycolysis during priming results in the inhibition of LPS-induced gene transcription of IL-1β (67).

Saturated fatty acid such as palmitate induced IL-1β secretion in LPS-treated primary macrophages, suggesting the activation of NLRP3 inflammasome by saturated fatty acids, of which mechanism involves the lysosomal rupture and cathepsin B release (68). Saturated fatty acids-induced activation of the NLRP3 inflammasome was mediated by intracellular crystallization accompanied with subsequent lysosomal dysfunction (69). Similarly, oxidized low-density lipoprotein that is recognized by scavenger receptor CD36, causes crystallization to induce the NLRP3 inflammasome activation (70). Oxidized phosphatidylcholine induced activation of the NLRP3 inflammasome mediated by miROS production and mitochondrial destabilization (71). In contrast, unsaturated fatty acids such as oleate and linoleate blocked IL-1β secretion induced by saturated fatty acids, or NLRP3 inducers such as nigericin, alum, and MSU in human monocytes/macrophages (72).

These show the link between the NLRP3 inflammasome and metabolic diseases, suggesting how the metabolic dysfunction leads to augmented inflammation. The activation of the NLRP3 inflammasome by metabolic stress involves a variety of cellular stress pathways including ROS production, ion fluxes, mitochondrial dysfunction, and lysosomal destabilization. The intracellular events involved in the activation of the NLRP3 inflammasome are interconnected and well-correlated to accomplish full activation of the NLRP3 inflammasome.

Collectively, despite of efforts to elucidate the upstream events during NLRP3 activation, a single unifying model has not been proposed, with the evidence obtained to date involving pharmacological inhibition rather than genetic approaches. Thus, it is difficult to analyze the indirect or off-target effects associated with NLRP3 activation. For example, it is difficult to ascertain whether mitochondrial dysfunction and mitochondria-derived ROS (mtROS) are the decisive factors for NLRP3 activation because other events such as ion flux changes and small organ damage, occur simultaneously. The intracellular signaling events may be interconnected or converge to further downstream steps to maximize NLRP3 inflammasome activation.

## PTM Regulation of the NLRP3 Inflammasome

**Figure 2:**
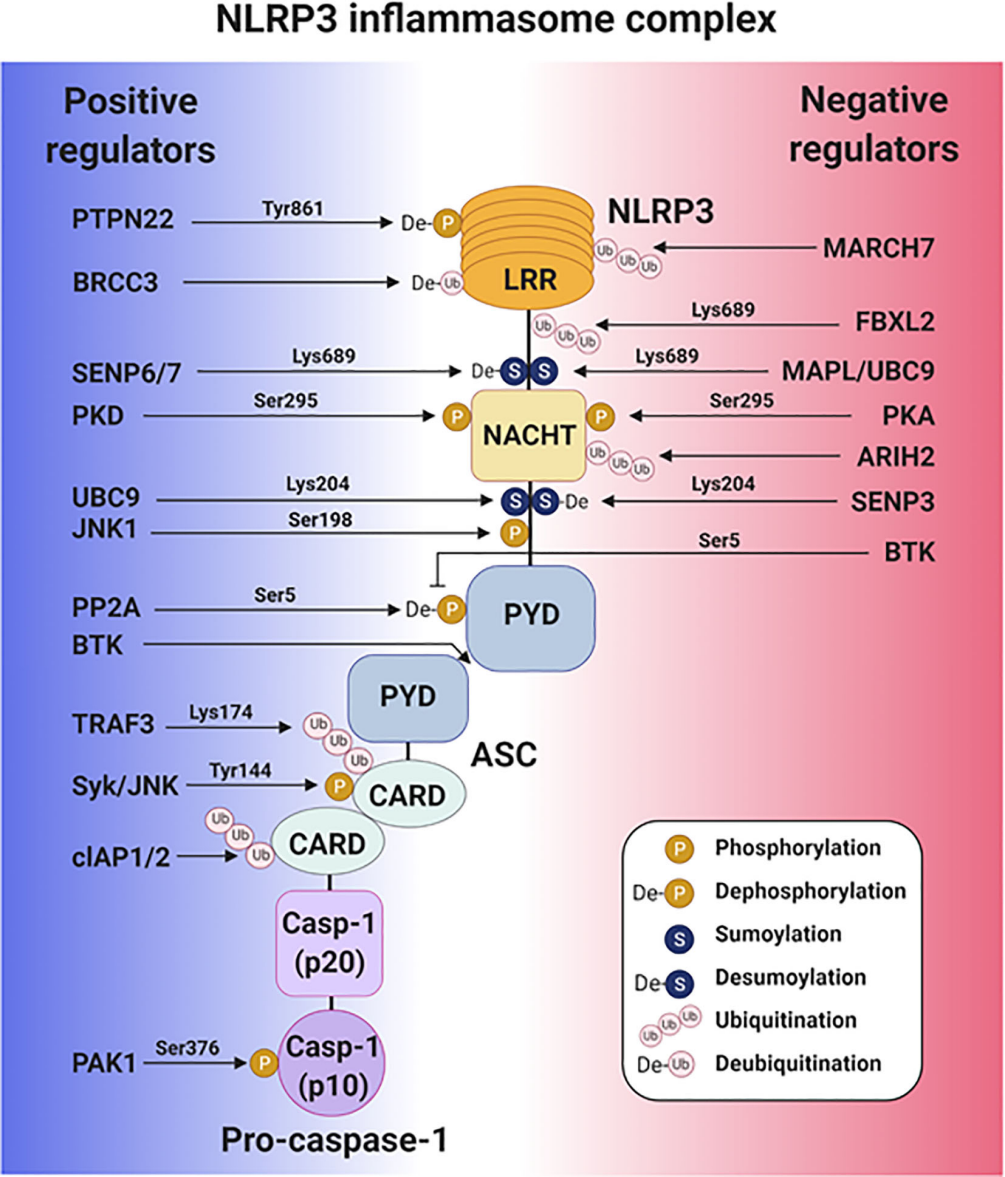
Post-translational regulation of the NLRP3 inflammasome activation. A schematic diagram of the intracellular proteins that positively and negatively regulate NLRP3 inflammasome activity.

Emerging roles have been identified for many intracellular molecules in regulating NLRP3 inflammasome activation. Some of the proteins promote NLRP3 inflammasome activation by binding to the inflammasome components to promote their PTMs, such as phosphorylation, sumoylation, and ubiquitination, while some prevent NLRP3 inflammasome activation *via* PTM. To manipulate the NLRP3 inflammasome for therapeutic purposes, identifying the intracellular proteins regulating PTM of the NLRP3 inflammasome and understanding their mechanisms of action is of crucial importance (**Figure 2**).

### Regulators of Phosphorylation

Phosphorylation of NLRP3 at Ser198 by JNK1 is an essential prerequisite event that occurs during the priming step, inducing the self-association of NLRP3 to promote active inflammasome assembly (73). Phosphorylation by JNK1 promotes NLRP3 deubiquitination, while S194A mutation or JNK inhibition interferes with the interaction of NLRP3 with BRCC3 and disrupts NLRP3 deubiquitination (73).

Protein kinase D (PKD) is required for NLRP3 inflammasome activation. PKD phosphorylates human Ser295 (mouse Ser291) in the NACHT domain of NLRP3 at the Golgi to free NLRP3 from mitochondria-associated endoplasmic reticulum membranes (MAMs), resulting in the recruitment of ASC to NLRP3 (74). Blockade of PKD activity led to the suppression of NLRP3 inflammasome activity in macrophages (74).

NIMA-related kinase 7 (NEK7) is a serine-threonine kinase that may be a crucial component for NLRP3 inflammasome activation. NEK7 interacts with NLRP3 to create ASC specks and induce oligomerization as an essential complex for caspase-1 activation (75). NEK7 can directly bind to NLRP3 protein, leading to NLRP3 inflammasome assembly due to K^+^ efflux. Moreover, chloride intracellular channel (CLIC)-dependent Cl^-^ efflux can promote NEK7-NLRP3 interactions and subsequent ASC oligomerization (43). However, it remains unclear how intracellular Cl^-^ efflux regulates the NEK7-NLRP3 interaction.

Syk and JNK are responsible for the phosphorylation of ASC in macrophages, with Tyr144 being a putative phosphorylation site (76). Interestingly, Syk has a cell type-specific role in NLRP3 inflammasome activation, as is not essential for this process in dendritic cells (76). The phosphorylation of Tyr144 in mouse ASC was shown to be required for ASC speck formation and caspase-1 activation in NLRP3 inflammasome activation but dispensable for the interaction of ASC with NLRP3 (76).

Protein kinase A (PKA) activation by prostaglandin E2 *via* the PGE2 receptor E-prostanoid 4 (EP4) leads to inhibitory phosphorylation of NLRP3 at Ser295 in human NLRP3, resulting in the suppression of ATPase activity of NLRP3 (77). In addition, PKA activation by bile acids through the TGR5 bile acid receptor leads to the phosphorylation of mouse NLRP3 at Ser 291, culminating in NLRP3 and ubiquitination the blockade of NLRP3 inflammasome activation (78).

Protein phosphatase (PP2A) participates in NLRP3 activation by dephosphorylating NLRP3 at Ser5 (79). Ser5 is located in the NLRP3 PYD-ASC PYD interaction motif, and its phosphorylation disrupts the association of NLRP3 and ASC (79). However, the kinase that phosphorylates Ser5 of NLRP3 has not been identified.

Protein tyrosine phosphatase nonreceptor type 22 (PTPN22) is required for NLRP3 inflammasome activation since PTPN22 knockdown results in decreased IL-1β secretion (80). PTPN22 promotes NLRP3 inflammasome activation by dephosphorylating Tyr861 in human NLRP3 (80). A Y861C mutation in NLRP3 has been reported in patients with chronic infantile neurologic cutaneous and articular syndrome (CINCA), and the Y861F mutation in NLRP3 has been shown to enhance NLRP3 inflammasome activation compared to wild-type NLRP3 (80).

Src homology 2 (SH2) domain-containing tyrosine phosphatase-2 (SHP2, encoded by the gene PTPN11) is a negative regulator of the NLRP3 inflammasome (81). SHP2 deficiency was shown to lead to excessive NLRP3 inflammasome activation in macrophages and aggravated peritonitis symptoms in a mouse model (81). SHP2 translocates to the mitochondria in response to NLRP3 inflammasome stimulators and binds to and dephosphorylates adenine nucleotide translocase 1 (ANT1), which plays a role in controlling mitochondrial permeability transition. SHP2 prevents the collapse of mitochondrial membrane potential and the subsequent release of mitochondrial DNA and reactive oxygen species, thereby suppressing hyperactivation of the NLRP3 inflammasome (81).

Bruton’s tyrosine kinase (BTK) has been reported to be a positive regulator of the NLRP3 inflammasome (82), as BTK inhibitors and dysfunctional BTK mutants inhibit NLRP3 inflammasome activation in macrophages, and BTK directly interacts with ASC and NLRP3, promoting ASC aggregation (82). The FDA-approved BTK inhibitor ibrutinib was shown to protect against ischemic brain injury by reducing mature IL-1β and caspase-1 activation in infiltrating macrophages and neutrophils in the infarcted area of the ischemic brain (82). Another study showed that a BTK inhibitor or BTK deficiency can impair NLRP3 inflammasome activation (83). BTK is associated with NLRP3 and ASC and promotes ASC speck formation and caspase-1 cleavage (83), with the BTK inhibitor ibrutinib shown to be effective in blocking IL-1β secretion in immune cells derived from Muckle-Wells syndrome patients (83). In contrast, a recent study showed a negative role of BTK in regulating NLRP3 inflammasome activity (84), with BTK deficiency enhancing NLRP3 inflammasome activity in macrophages (84). BTK binds to NLRP3 during the priming step of inflammasome activation, preventing NLRP3 inflammasome assembly induced by NLRP3 activators during the activation phase of inflammasome activation (84). BTK was shown to block PP2A-mediated dephosphorylation of Ser5 in the pyrin domain of NLRP3 to inhibit the NLRP3 inflammasome (84). However, the exact role of BTK in NLRP3 inflammasome regulation and whether BTK has different roles in different contexts needs to be further examined.

TGF-β activated kinase-1 (TAK1) has been suggested to function as a negative regulator to prevent spontaneous NLRP3 activation (85). TAK1 deficiency was shown to lead to spontaneous NLRP3 inflammasome activation in macrophages, suggesting that TAK1 maintains NLRP3 inflammasome quiescence (85).

p21-activated kinase 1 (PAK1) is involved in caspase-1 activation induced by *Helicobacter pylori* LPS (86) by phosphorylating Ser376 in the p10 subunit of caspase-1 (86).

### Regulators of Sumoylation

NLRP3 was conjugated with SUMO-2/-3 and sumoylated at basal state, mediated by interaction between MAPL (also known as MUL1), a SUMO E3 ligase, and NLRP3 (87). Activating signals such as nigericin reduced sumoylated NLRP3, possibly by disrupting NLRP3-MAPL interaction (87). The SUMO E2 ligase, UBC9 interacted with the SUMO consensus motif surrounding K689 of NLRP3 (87). Deficiency of sentrin-specific protease 6 (SENP6) and SENP7, which are SUMO de-conjugating enzymes, reduced NLRP3 inflammasome activation (87). In contrast, SUMO1-catalyzed sumoylation of NLRP3 Lys204 promoted inflammasome activation whereas SENP3, a deSUMOylase, induced NLRP3 deSUMOylation to attenuate inflammasome activation (88). The precise role of sumoylation by different enzymes and at different activation steps needs to be further elucidated.

### Regulators of Ubiquitination

Pellino2, an E3 ubiquitin ligase, is required for NLRP3-induced ASC oligomerization and mature IL-1β production (89). LPS induces the interaction of Pellino2 with NLRP3 and Pellino2 FHA, and RING domains facilitate NLRP3 activation by promoting the K63-linked ubiquitination of NLRP3 during the priming step (89). Pellino2-deficient mice and myeloid cells show impaired NLRP3 activation in response to Toll-like receptor priming, NLRP3 stimuli and bacterial challenge (89).

BRCA1-BRCA2 containing complex subunit 3 (BRCC3) plays a role in NLRP3 inflammasome *via* deubiquitination of the LRR domain of NLRP3 by directly binding to ubiquinated NLRP3 (90). Furthermore, BRCC3 knockdown was shown to reduce the ATP-induced secretion of mature IL-1β by in macrophages (91).

Deficiency of tumor necrosis factor alpha-induced protein 3 (TNFAIP3, also known as A20), a deubiquitinating enzyme, was observed to enhance NLRP3 inflammasome-mediated caspase-1 activation, pyroptosis, and IL-1β secretion in macrophages, while activation of the NLRC4 and AIM2 inflammasomes was not affected (92).

The E3 ubiquitin ligase TRIM31 plays a negative regulatory role in NLRP3 inflammasome activation (93). TRIM31 directly binds to NLRP3, promoting the K48-linked polyubiquitination and proteasomal degradation of NLRP3 (93). Furthermore, TRIM31 deficiency enhances IL-1β secretion *in vivo* and aggravates alum-induced peritonitis in mice (93).

FBXL2 interacts with Trp73 in NLRP3 and targets Lys689 in NLRP3 for ubiquitin ligation, leading to the degradation of NLRP3 and a reduction in IL-1β and IL-18 secretion in human inflammatory cells (8).

The E3 ubiquitin ligase MARCH7 is involved in the ubiquitination and degradation of NLRP3 in neurotransmitter dopamine-treated macrophages (94). Dopamine negatively regulates the NLRP3 inflammasome *via* cyclic adenosine monophosphate (cAMP), which binds to NLRP3 and promotes its K48-linked polyubiquitination and degradation through MARCH7 activity (94). In addition, it was demonstrated that the LRR domain of NLRP3 is a key region for MARCH7-mediated ubiquitination and degradation (94).

Ariadne homolog 2 (ARIH2), an E3 ligase, interacts with the NACHT domain of NLRP3, leading to the K48- and K63-linked ubiquitination of NLRP3 and downregulation of NLRP3 inflammasome activation (95). ARIH2 deficiency results in increased NLRP3 inflammasome activation and IL-1β production, while ARIH2 overexpression inhibits NLRP3 inflammasome activation (95).

TNFR-associated factor 3 (TRAF3) is a direct E3 ubiquitin ligase for ASC (96), with ASC ubiquitination at Lys174 being crucial for speck formation and NLRP3 inflammasome activation. TRAF3 deficiency results in impaired ASC ubiquitination and cytosolic aggregate formation, resulting in decreased inflammasome responses during RNA virus infection (96).

HOIL-1L is a component of the linear ubiquitination assembly complex (LUBAC), which consists of HOIL-1L, HOIP, and Sharpin. ASC is linearly ubiquitinated by LUBAC, and HOIL-1L is required for ASC foci formation and NLRP3/ASC inflammasome assembly (97). Furthermore, HOIL-1L-deficient macrophages were observed to be impaired in mature IL-1β secretion upon NLRP3 activation stimuli (97).

Inhibitors of apoptosis proteins (IAPs), such as cIAP1 and cIAP2, have E3 ubiquitin ligase activity. cIAP1 and cIAP2 interact directly with caspase-1 and are required for inflammasome assembly and caspase-1 activation, which is mediated by the K63-linked polyubiquitination of caspase-1 (98).

Collectively, PTMs of the NLRP3 inflammasome show that the NLRP3 inflammasome activity is regulated by various signaling components in a complex and elaborated fashion. It needs to be further elucidated that how each PAMP or DAMP modulates the activity of PTM-related signaling molecules and what the interactions between different PTMs are.

## Small Molecules and Phytochemicals Regulating the NLRP3 Inflammasome Activation

The NLRP3 inflammasome plays a pivotal role in development and progress of many common inflammatory diseases (99). The NLRP3 inflammasome has been implicated in the pathogenesis of metabolic disorders such as type 2 diabetes (100), atherosclerosis (101), obesity (102), and gout (103). In addition, the role of NLRP3 is noted to contribute to the pathology of central nervous system diseases including Alzheimer’s disease (104) and Parkinson’s disease (105). Abnormal activation of the NLRP3 inflammasome is associated with intestinal cancer and auto-inflammatory diseases such as keratitis/conjunctivitis (106, 107). Therefore, the discovery of pharmacological inhibitors targeting NLRP3 inflammasome components provides a novel strategy for the development of potential therapeutics in a wide range of human diseases (**Table 1**).

**Table 1:** Small molecules and phytochemicals regulating the NLRP3 inflammasome activation.

Agents		Target	Mechanism	References
Synthetic small molecules	3,4-Methylenedioxy-β-nitrostyrene	NLRP3 NACHT and LRR domains	Blocks NLRP3 ATPase activity	(114)
	Bay 11-7082	Kinase activity of IKKβ and the NLRP3 NATCH domain	Inhibits the NF-κB pathway and prevents NLRP3 function	(113)
	CY-09	NLRP3 NATCH domain	Directly interacts with NLRP3 and inhibits cystic fibrosis transmembrane conduction regulator (CFTR) channels	(108)
	G5	NLRP3 (indirectly)	Inhibits deubiquitination of NLRP3	(91)
	Glyburide, 16673-34-0, JC124	NLRP3 (indirectly)	Inhibits ATP sensitive K+ channels downstream of P2X7 and ASC aggregation	(117–119)
	MCC950	NLRP3 NATCH domain	Blocks the ability of NLRP3 to hydrolyzed ATP	(109)
	OLT1177	NLRP3 NATCH domain	Inhibits NLRP3 ATPase activity by directly binding to the NLRP3 ATPase region.	(110)
	Tranilast	NLRP3 NATCH domain	Prevents NLRP3-NLRP3 interaction and increases K63‐linked ubiquitination of NLRP3	(115)
	VX-740, VX-765	Caspase-1	Inhibits caspase-1 activity	(120, 121)
Phytochemicals	β-carotene	NLRP3 PYD domain	Inhibits the NLRP3 inflammasome by directly binding to the pyrin domain (PYD) of NLRP3	(122)
	CAPE	ASC PYD domain	Blocks NLRP3-ASC interactions by directly binding with ASC	(123)
	Celastrol	NLRP3 (indirectly)	Inhibits NLRP3 inflammasome activation induced by ATP, nigericin, and ox-LDL	(124, 125)
	EGCG	NLRP3 (indirectly)	Inhibits NLRP3 inflammasome by blocking mitochondrial DNA synthesis and ROS production	(126)
	Licochalcone A	NLRP3 (indirectly)	Inhibits ASC speck formation and mitochondrial ROS	(127)
	Sulforaphane	NLRP3 (indirectly)	Induces autophagy resulting in the suppression of the NLRP3 inflammasome	(128)

### Synthetic Small Molecules

Among the synthetic small molecules, CY-09 (108), MCC950 (109), and OLT1177 (110) bind directly to the NATCH domain of NLRP3 and block NLRP3 ATPase activity. CY-09 is a CFTR (inh)-172 (C172) analog that inhibits cystic fibrosis transmembrane conduction regulator (CFTR) channels by directly interacting with the NLRP3 Walker A motif of NLRP3 NACHT domain and disrupting ATP binding to NLRP3 (108). CY-09 has shown excellent prophylactic and therapeutic properties in mouse models of gout, type 2 diabetes, and Cryopyrin-associated periodic syndrome (CAPS) (108). MCC950 inhibits both canonical and noncanonical NLRP3, but not AIM2, NLRC4 or NLRP1 activation, in mouse macrophages, human monocyte derived macrophages, and human peripheral blood mononuclear cells (111). MCC950 treatment was effective to attenuate inflammatory symptoms of NLRP3-related diseases including experimental autoimmune encephalomyelitis and CAPS (111). MCC950 directly binds to the Walker B motif in the NLRP3 NACHT domain, inhibiting ATPase activity and NLRP3 inflammasome formation (109). OLT1177 is an active β-sulfonylnitrile compound that has passed phase 1 clinical trials for the treatment of degenerative arthritis and is currently being evaluated in phase 2 clinical trials (112). OLT1177 has been shown to block both standard and nonstandard NLRP3 inflammasome activation and ATPase activity by directly binding to NLRP3 (110). Bay 11-7082 binds to NLRP3 through alkylation of cysteine residues in the NLRP3 ATPase region, thereby blocking NLRP3 inflammasome function in addition to its inhibition of the kinase activity of IKKβ (113). 3,4-Methylenedioxy-β-nitrostyrene directly binds to NACHT and LRR domains of NLRP3, blocking its ATPase activity (114). Tranilast, a tryptophan metabolite analog, binds to NACHT domain of NLRP3, preventing NLRP3-NLRP3 interaction and alleviating the symptoms of gouty arthritis, cryopyrin-associated autoinflammatory syndromes, and type 2 diabetes (115). In addition, tranilast increases K63-linked ubiquitination of NLRP3 (116).

Glyburide (99, 117) inhibits NLRP3 inflammasome activity. The small molecule 16673-34-0 (118), an intermediate substrate produced during the synthesis of glyburide and the low-molecular-weight JC124 (119), generated during the structural optimization of glyburide, alleviates the side effects of glyburide.

VX-740 (Pralnacasan) (120) and its analog VX-765 (121) are peptidomimetic inhibitors of caspase-1 and inhibit the proinflammatory cytokines IL-1β and IL-18.

G5 (3,5-bis[(4-Nitrophenyl)methylene]-1,1-dioxide,tetrahydro-4H-thiopyran-4-one), a small molecule inhibitor of deubiquitination, inhibits NLRP3 inflammasome activation induced by ATP and nigericin in LPS-primed macrophages (91). G5 promotes ubiquitination of NLRP3 NACHT and LRR domains with mixed K63 and K48 ubiquitin chains, resulting in the blockade of NLRP3 inflammasome activation (91).

As excessive NLRP3 inflammasome activation has been shown to be closely associated with the pathophysiology of a wide array of disorders, synthetic small molecules that directly or indirectly inhibit the NLRP3 inflammasome have been suggested as promising therapeutic agents (**Table 1**). The specificity of the target sites with high potency would be the critical prerequisite requirement to develop the new NLRP3 inflammasome inhibitors.

### Phytochemicals

Phytochemicals are substances synthesized by medicinal plants and extracted for therapeutic purposes (Katz and Baltz, 2016). Plants are a preferred therapeutic substance because they are easily accessible and cost-effective, and there are many investigations on phytochemicals that modulate the inflammatory response by regulating specific NLRP3 inflammasome components. Phytochemicals that directly or indirectly regulate the NLRP3 inflammasome include β-carotene (122), caffeic acid phenethyl ester (CAPE) (123), sulforaphane (128), celastrol (124, 125), epigallocatechin-3-gallate (EGCG) (126), and licochalcone A (127).

β-Carotene binds directly to pyrin domain (PYD) of NLRP3 (122). Molecular modeling and mutation study showed that β‐carotene interacted with Ala69, Val72, Trp73, Tyr84, and Glu91 in the hydrophobic groove of H5 and H6 (122). β-Carotene inhibits ATP, MSU crystals, and nigericin-induced NLRP3 inflammasome activation in macrophages. In addition, β-carotene was shown to decrease IL-1β secretion by human synovial cells isolated from gout patients, demonstrating the potential inhibitory effect of β-carotene on human gout (122).

CAPE directly binds to PYD of ASC, leading to the disruption of the NLRP3-ASC association as demonstrated by SPR analysis and pull-down experiment (123). In a murine gouty arthritis model, the oral administration of CAPE was shown to attenuate inflammatory symptoms by inhibiting MSU crystal-induced caspase-1 activation and IL-1β production in air pouch exudate and foot tissue (123). To our best knowledge, CAPE is the only phytochemical known to bind directly to ASC.

Sulforaphane (SFN) regulated upstream signaling pathways of AMP-activated protein kinase-autophagy axis to suppress the NLRP3 inflammasome activation. SFN induced autophagosome formation and p62 degradation in hepatocytes, whereas it inhibited the activation of mammalian target of rapamycin (mTOR), a negative regulator of autophagy, suggesting that SFN promotes autophagy in hepatocytes (128). SFN induced phosphorylation of AMP-activated protein kinase and consequent activation of autophagy, resulting in inhibition of the NLRP3 inflammasome in the liver. Oral administration with SFN prevented non-alcoholic fatty liver disease symptoms in mice fed high fat diet by inhibiting the NLRP3 inflammasome in the liver (128). In addition, SFN can effectively alleviate acute gouty inflammation by inhibiting NLRP3 inflammasome activation induced by MSU crystals in in macrophages (129). The inhibition of NLRP3 inflammasome activation by SFN is independent of the reactive oxygen species pathway in macrophages (129). These show that the regulation of autophagy can be used as a beneficial strategy to prevent the NLRP3 inflammasome activation.

There are phytochemicals affecting intracellular events of the NLRP3 inflammasome, including K^+^ efflux, mitochondrial ROS production, and mitochondrial DNA release. Celastrol suppresses the K^+^ efflux induced by ATP and nigericin in primary macrophages, resulting in suppression of the NLRP3 inflammasome (124, 125). Celastrol pretreatment reduced the ability of ATP-stimulated macrophages to promote cancer cell migration and invasion (124, 125). The inhibitory effects of licochalcone A and EGCG were mediated through the regulation of mitochondrial dysfunction. Licochalcone A blocked mitochondrial ROS generation induced by *Propionibacterium acnes (P. acnes*) and rotenone in mouse primary macrophages (127). Similarly, EGCG prevented production of ROS induced by NLRP3 activators such as MSU, ATP, and nigericin and suppressed *de novo* synthesis of mitochondrial DNAs induced by MSU in primary mouse macrophages (126). The specific target molecules or mechanisms by which these phytochemicals modulate mitochondrial function remain to be further investigated.

There are a few phytochemicals directly binding to the NLRP3 inflammasome components while others work indirectly by modulating upstream signaling pathways of the NLRP3 inflammasome (**Table 1**). Elucidating the exact action mechanism by which the phytochemicals exert the inhibitory activity would provide an important information on specific targets to be regulated and drug discovery strategies for novel pharmacological inhibitors.

## Conclusion

With the lifespan improvements made possible by modern medical developments, the occurrence of metabolic and age-related diseases is increasing. Previous studies have demonstrated that the NLRP3 inflammasome is a key mediator in the development of various metabolic diseases and host inflammatory responses. The discoveries of NLRP3 inflammasome modulators, such as NEK7 and GSDMD, demonstrate significant advances in this field.

The PTM regulation of the NLRP3 inflammasome components can be potential targets for the development of specific drugs or inhibitors of the NLRP3 inflammasome. However, unidentified PTM sites of NLRP3 and PTM-related enzymes remain, and the correlations and interactions between the various types of PTMs of the NLRP3 inflammasome components require further investigation. As research on NLRP3 activation becomes more important, the targeting of NLRP3 as a therapeutic strategy for many diseases is rapidly progressing. The current treatment for NLRP3-related pathologies is to indirectly or directly inhibit the activation of the NLRP3 inflammasome using pharmacological inhibitors. NLRP3 inhibitors function as effective therapeutic agents for many inflammatory disorders by inhibiting the pro-inflammatory cytokines IL-1β and IL-18 and maintaining cellular homeostasis.

In summary, understanding how intracellular proteins, PTMs, and small molecule inhibitors regulate the NLRP3 inflammasome activation will provide crucial information for elucidating the host defense mechanisms in response to pathogen and tissue damage and in constructing effective therapeutic strategies for chronic diseases.

## Author Contributions

JS performed the acquisition of the data and information, and mainly wrote a draft of the manuscript. HK and Y-YC provided the data and information and participated in writing a draft of the manuscript. HL critically evaluated the content and participated in writing a draft of the manuscript. JL constructed the idea of the content, performed the acquisition of information, and wrote and finalized the manuscript. All authors contributed to the article and approved the submitted version.

## Funding

This study was supported by grants from the National Research Foundation of Korea (NRF‐2019R1A2C2085739, NRF-2019R1I1A1A01056377, NRF-2020R1A4A2002894), funded by the Korean government (Ministry of Science, ICT and Future Planning).

## Conflict of Interest

The authors declare that the research was conducted in the absence of any commercial or financial relationships that could be construed as a potential conflict of interest.
